# Supplementary figures and images for: A BRC1-modulated switch in auxin efflux accounts for the competition between Arabidopsis axillary buds
Source: PLoS Biol. 2025 Sep 15;23(9):e3003395. doi: 10.1371/journal.pbio.3003395 (PMC12456834; doi:10.1371/journal.pbio.3003395)

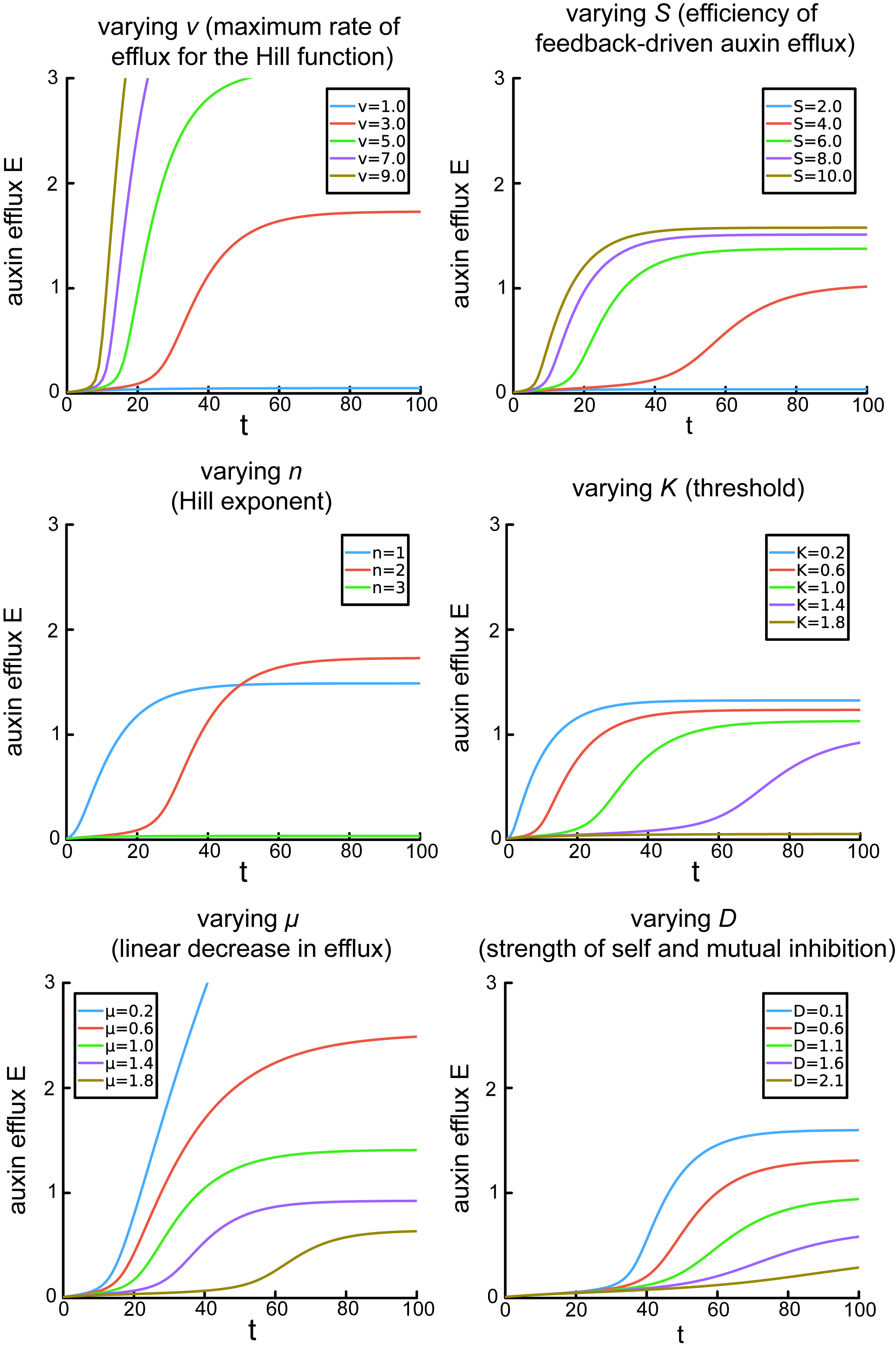

Supplement: S1 Fig — Each panel shows the auxin efflux E over time obtained from 5 deterministic simulations performed until time step t = 100, changing only one parameter value. Because these are deterministic simulations, the auxin efflux F is identical to that of E. Scripts of simulations underlying this figure can be found at doi 10.17863/CAM.120831. (TIF) [file pbio.3003395.s001.tif]

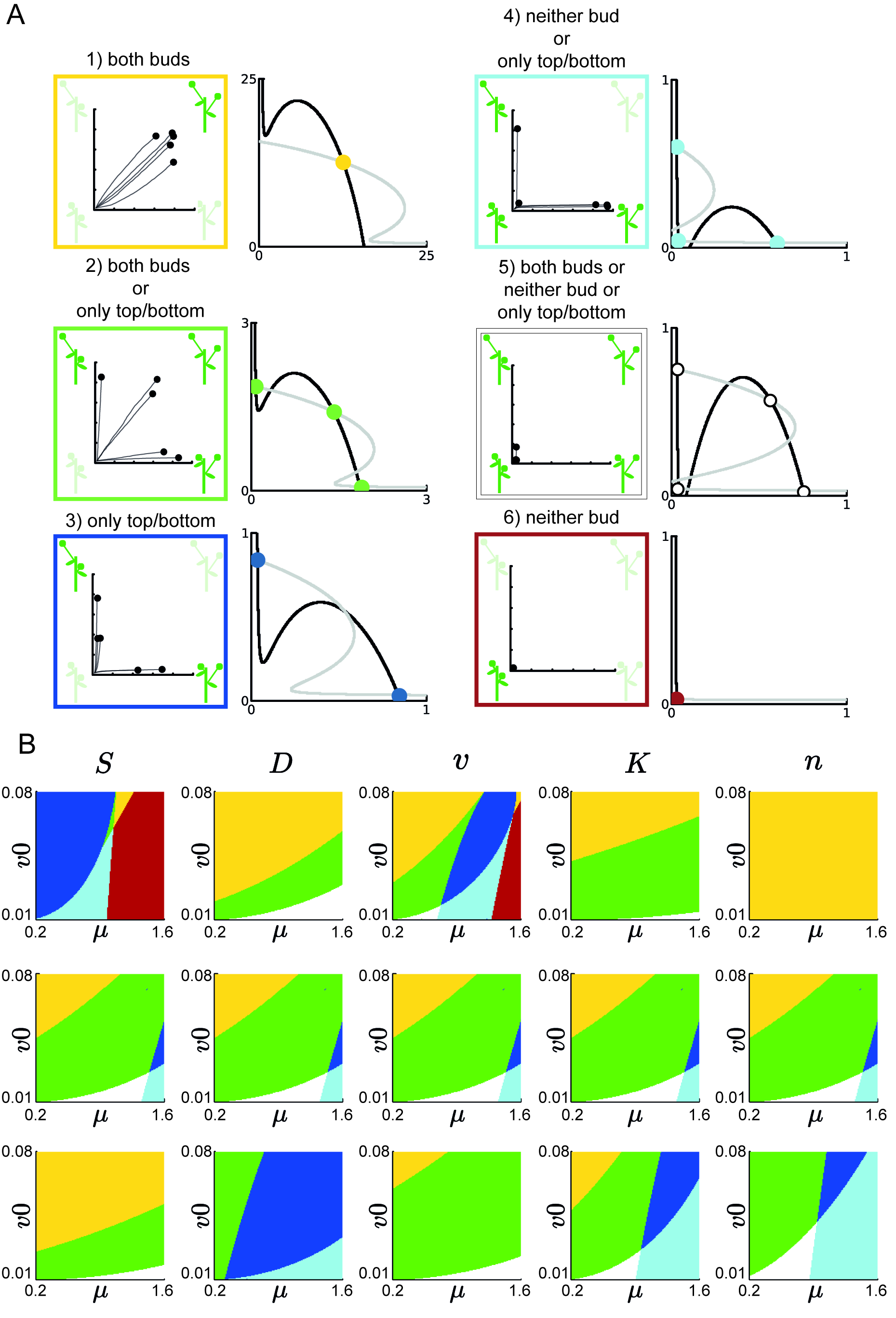

Supplement: S2 Fig — (A) The six possible behaviors found across parameter space, illustrated with a Mitchison plot of five simulations in the corresponding area of parameter space. The behaviors are categorized based on the number of stable steady states and their values. Corresponding nullclines in black and grey show how the shape of the nullclines influences the number of stable steady states, with steady states indicated by a dot at the intersection of the nullclines. Each behavior is represented in a different color: yellow represents cases where both buds activate, green when either both buds activate or only one bud activates, navy when only one bud activates, light blue when either neither bud activates or only one bud, white when all four outcomes are possible (neither bud, both buds, or only one bud), and red when neither bud activates. (B) Color maps showing how the (v0, µ) slice chosen for further analysis (middle row) is affected by changes in S, D, v, K and n. The behavior at each point in parameter space on the color map is indicated by the color of the pixel at that point, as specified in panel A. The middle slice was chosen based on its ability to deliver core features of bud activation. The slices above and below show how a respective 50% decrease or increase in each parameter value changes the growth outcomes. For example, at lower D there is a larger area of parameter space where both buds grow (behavior 1 – yellow). Here, colors are assigned based on the theoretically possible behaviors, as determined by the number and values of stable steady states. In Figs 4 and S3, colors are assigned based on the bud growth outcomes that were obtained after running 100 stochastic simulations for 120 time steps, for each combination of (v0, µ) values. Scripts of simulations underlying this figure can be found at doi 10.17863/CAM.120831. (TIF) [file pbio.3003395.s002.tif]

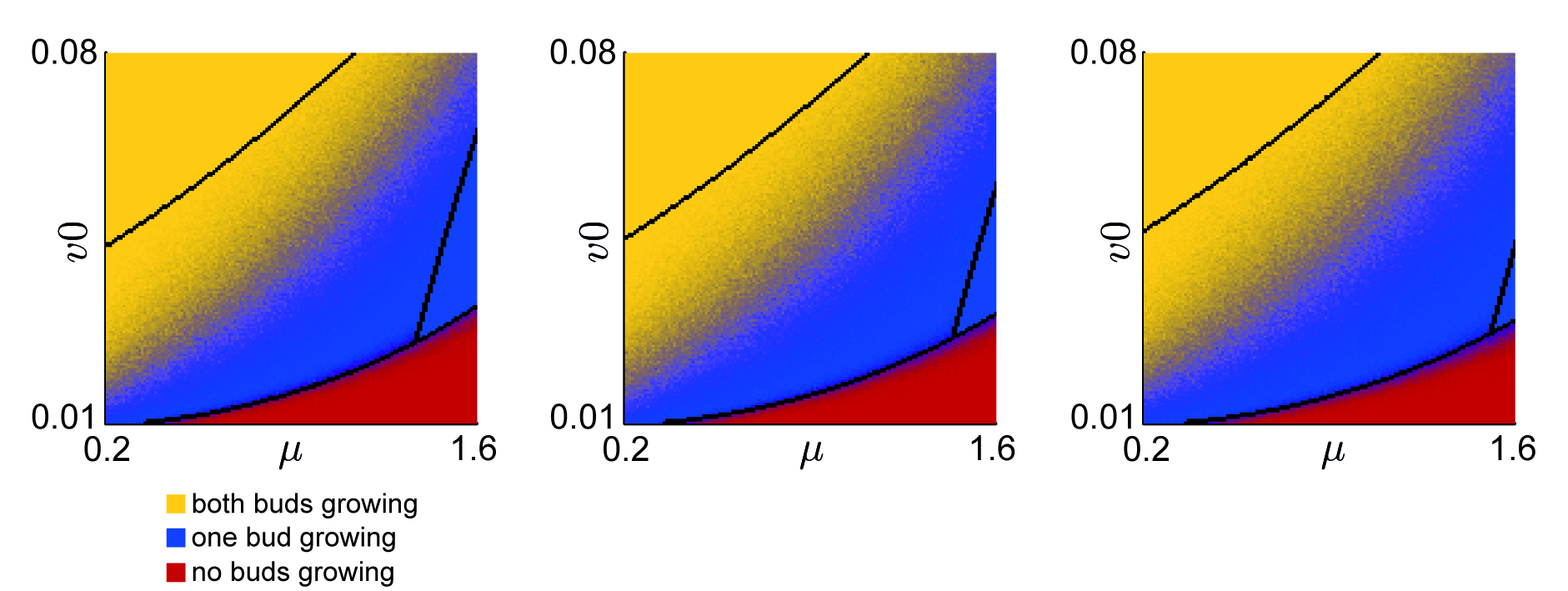

Supplement: S3 Fig — The stochastic color map from Fig 4B (center) is shown with a 5% decrease (left) and a 5% increase (right) applied to parameters S, D, v, K, Q, and η. Scripts of simulations underlying this figure can be found at doi 10.17863/CAM.120831. (TIF) [file pbio.3003395.s003.tif]

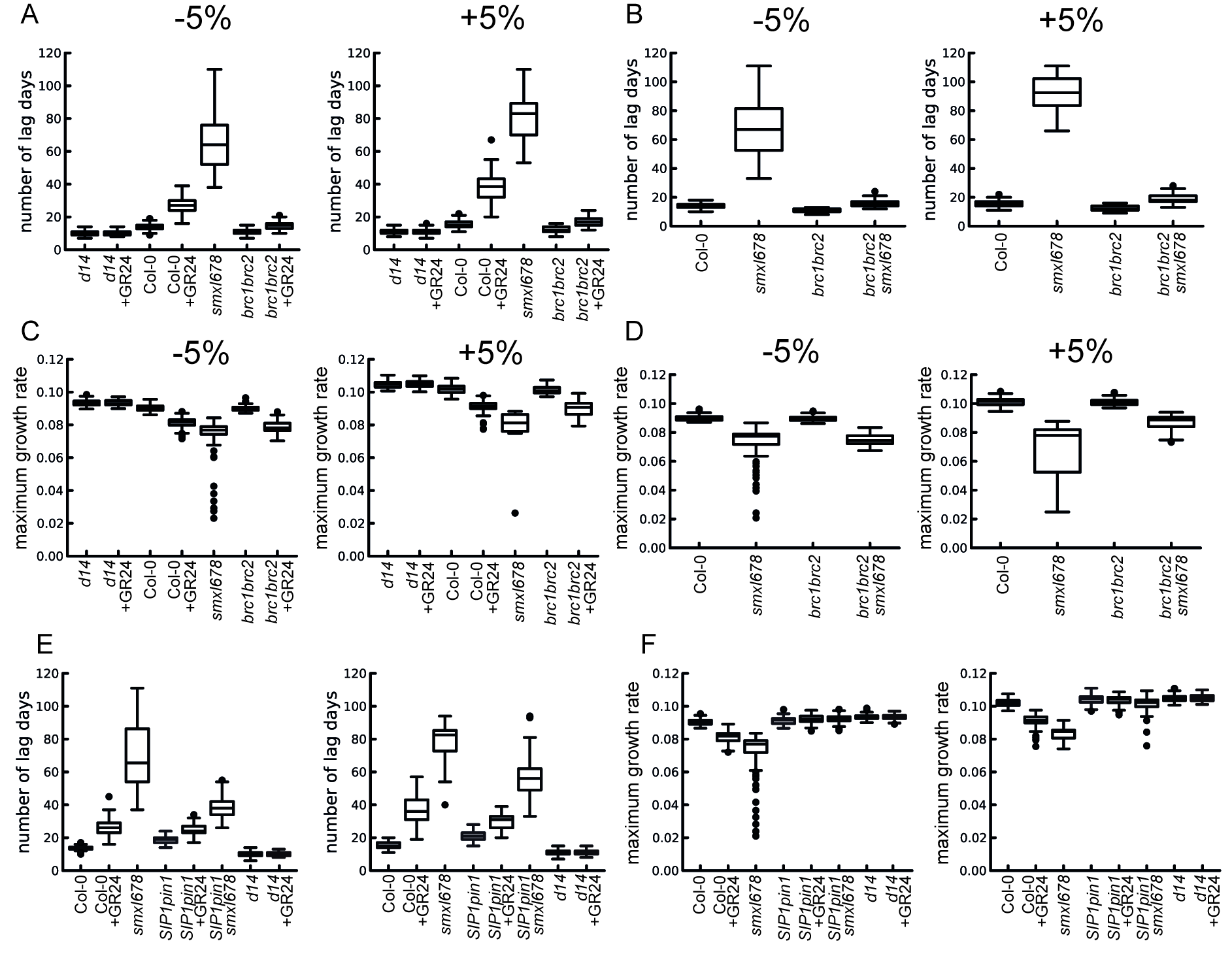

Supplement: S4 Fig — In each case, the number of lag days and maximum growth rate was calculated from 100 simulations performed for 120 time steps, with all parameter values increased or decreased by 5%, as compared to the plots in the main text. Scripts of simulations underlying this figure can be found at doi 10.17863/CAM.120831. (TIF) [file pbio.3003395.s004.tif]

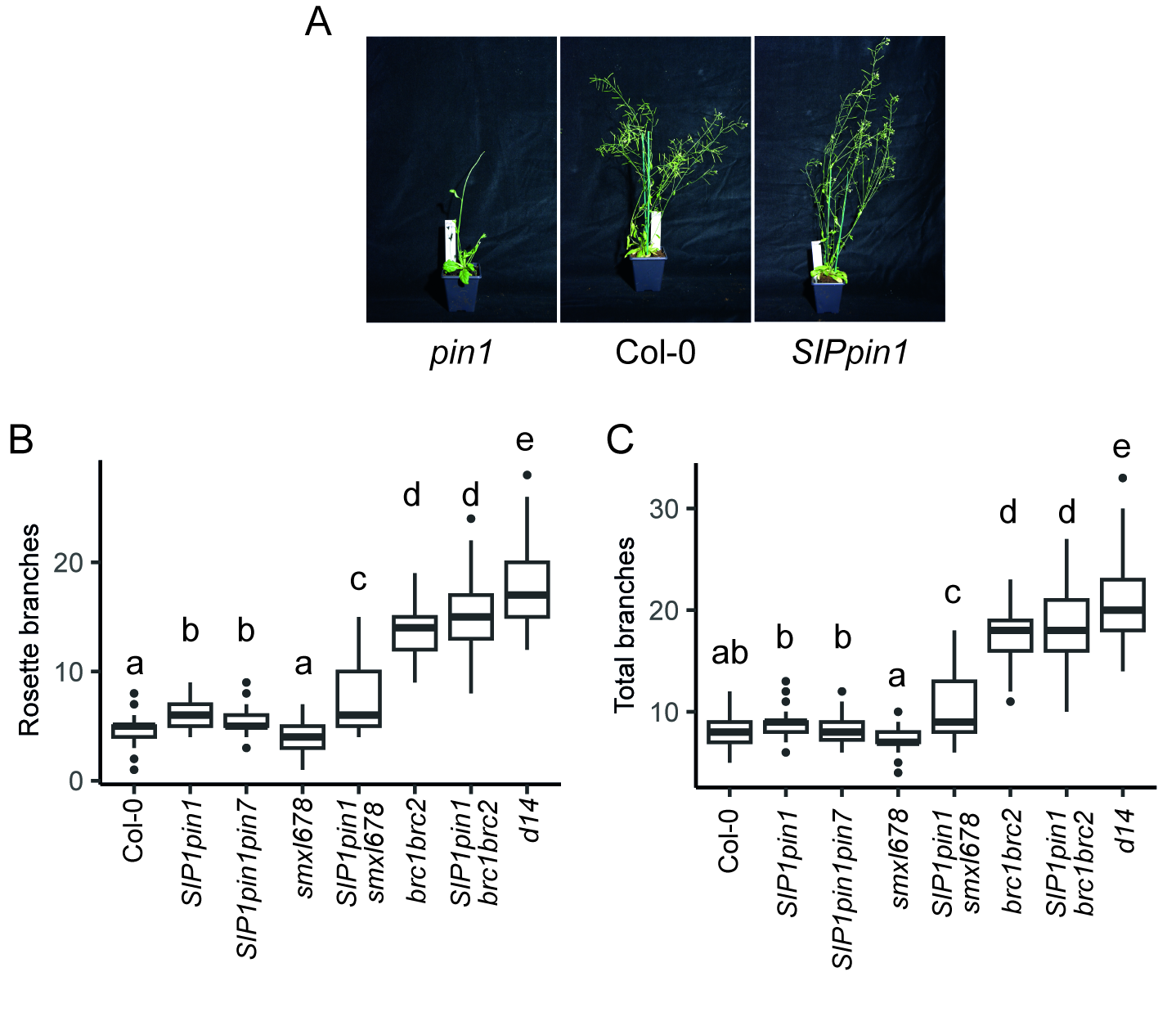

Supplement: S5 Fig — (A) Photograph of a representative plant from the genotypes indicated, taken at terminal flowering. (B) Primary rosette and (C) total (cauline and rosette) branch number at terminal flowering in plants of the genotypes indicated grown under long day conditions, n = 53–68. The boxes span the first to third quartile, the line represents the median. The whiskers indicate the variability outside the upper and lower quartiles. A generalized linear multi-level model was used to assess the effect of genotype while accounting for variability across experiments. Comparisons were adjusted using Bonferroni correction. Different letters indicate statistically significant differences at p < 0.05. Data and analysis underlying this figure can be found at doi 10.17863/CAM.120831. (TIF) [file pbio.3003395.s005.tif]

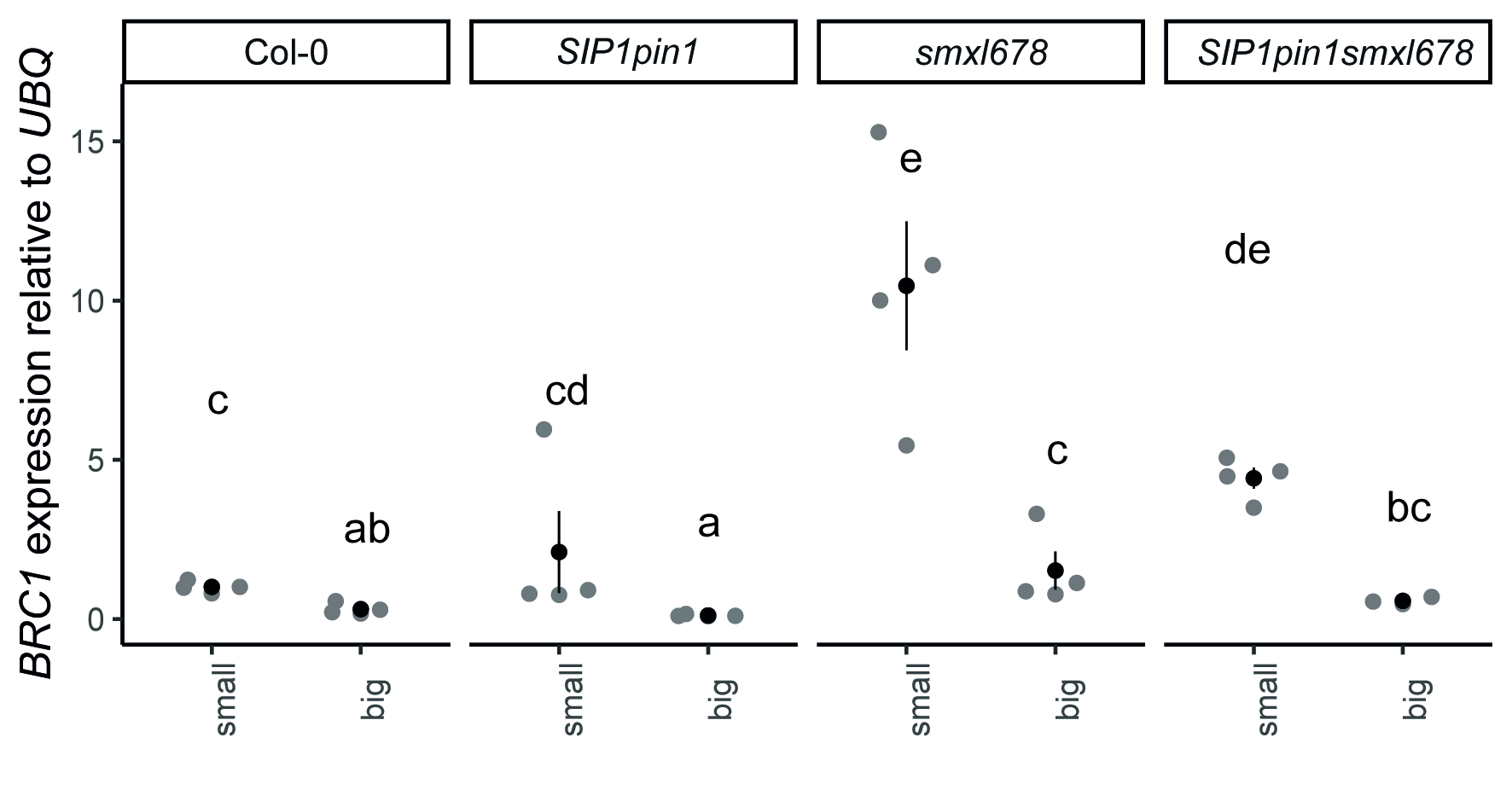

Supplement: S6 Fig — Buds were harvested from cauline nodes of whole Arabidopsis plants. Each point represents a biological replicate, with the superimposed mean and associated standard error. Each biological replicate corresponds to the expression level measured in at least 10 pooled buds. Statistical comparisons were made on the log transformed data, using two-way ANOVA with Bonferroni correction. Different letters indicate statistically significant differences at p < 0.05. Data and analysis underlying this figure can be found at doi 10.17863/CAM.120831. (TIF) [file pbio.3003395.s006.tif]

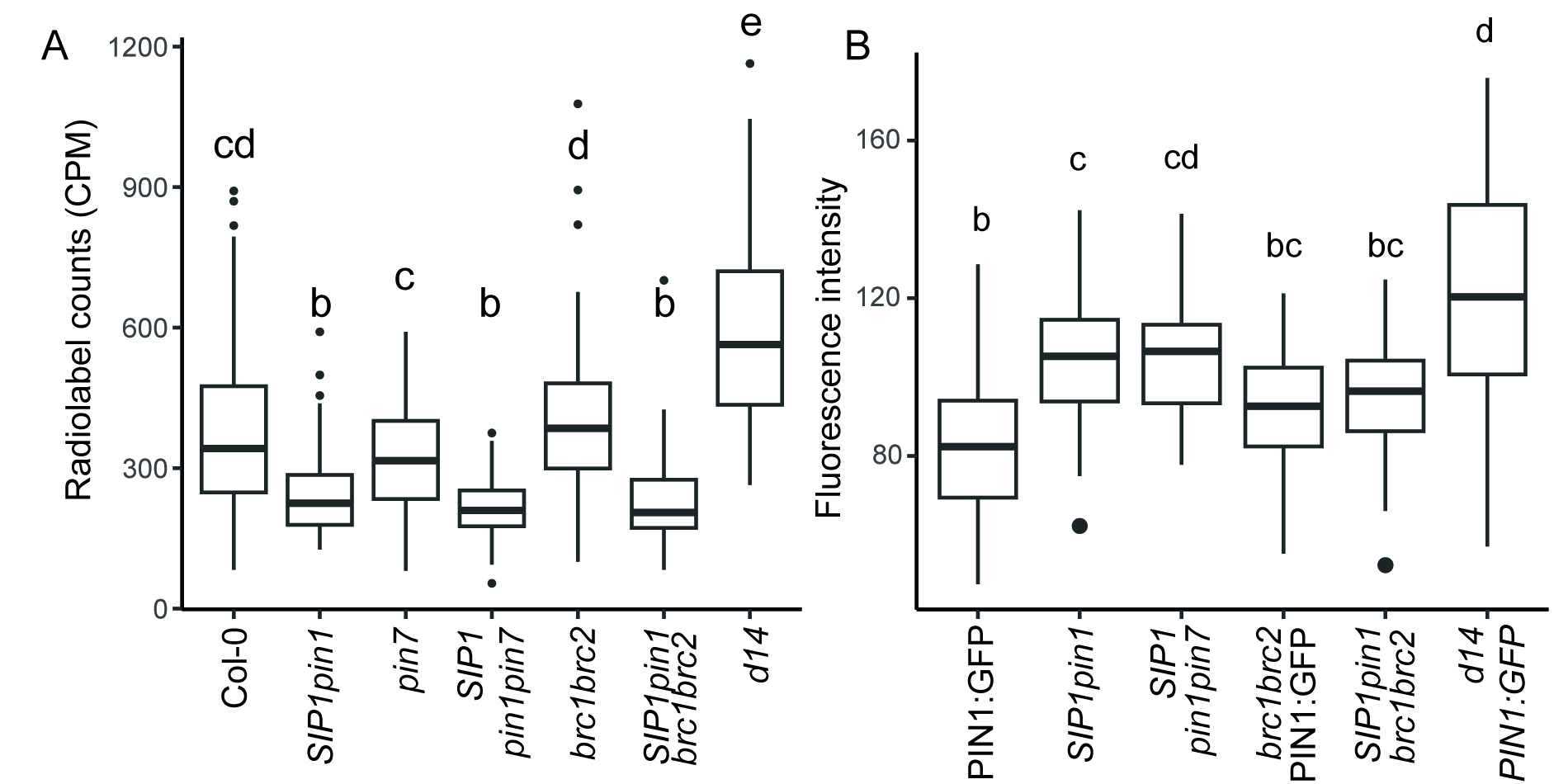

Supplement: S7 Fig — (A) Bulk auxin transport through 15 mm basal inflorescence stem segments of 6–8 week old plants of the genotypes indicated. Transport was determined as accumulation of 3H auxin in the basal 5 mm of stem after 18 h of incubation of the apical end 22 nM 3H-IAA, n = 57–68. (B) Boxplot of the mean fluorescence intensity of basal plasma membranes of xylem parenchyma cells from at least 8 stems per genotype. For each stem, the mean fluorescence intensity represents the mean of the 5 brightest membranes for that stem. Fluorescence intensity was extracted using the ZEN imaging software, n = 9–37 stems. Letters indicate statistically significant differences at p < 0.05 from a multi-level model with Bonferroni correction. Data and analysis underlying this figure can be found at doi 10.17863/CAM.120831. (TIF) [file pbio.3003395.s007.tif]

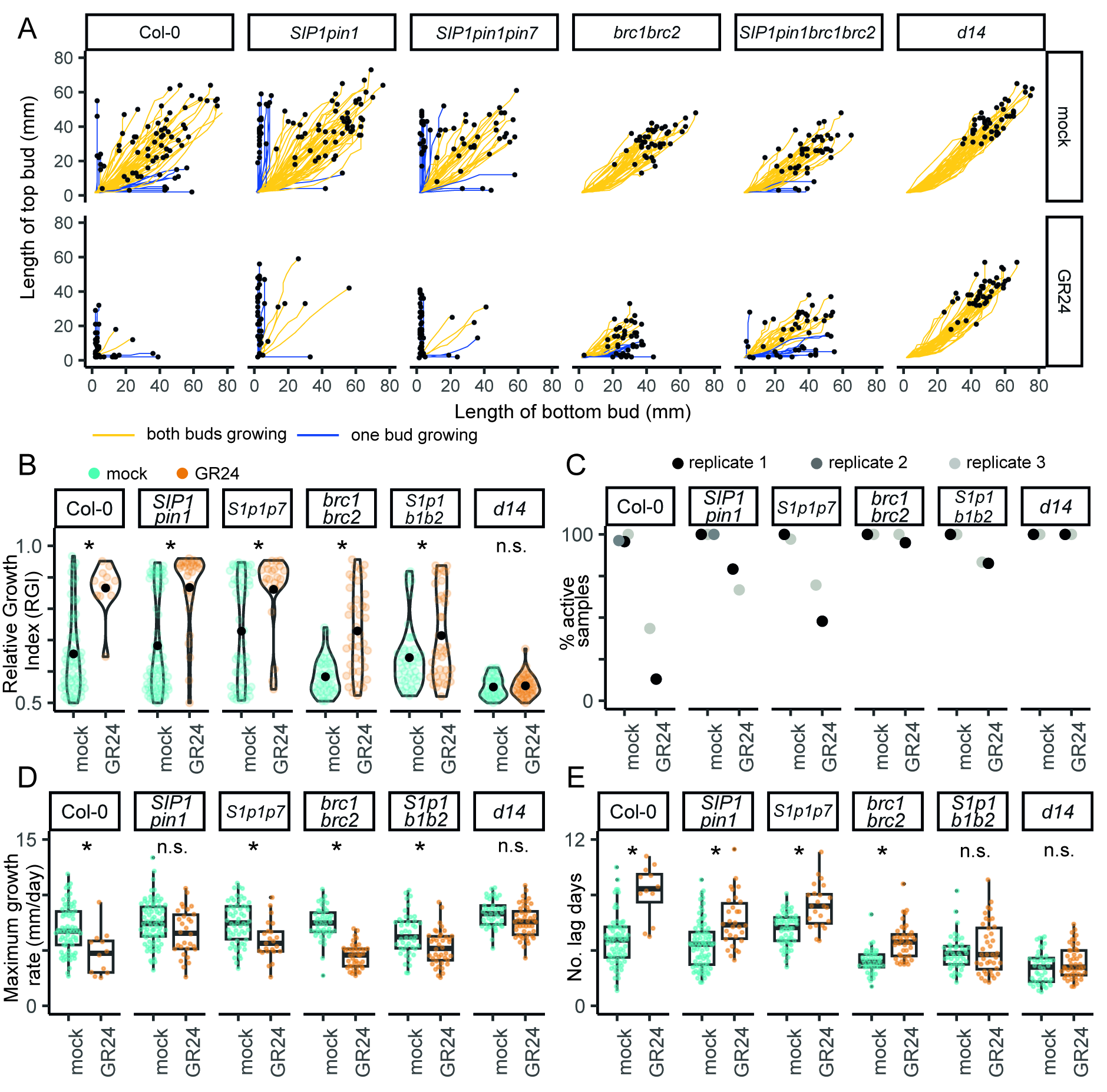

Supplement: S8 Fig — (A) Mitchison plot of 2-node explants from two-three experimental replicates of Col-0, SIP1pin1, SIP1pin1pin7, brc1brc2, SIP1pinbrc1brc2, and d14, with basal mock or 5 µM GR24 treatment. Buds were measured daily for 12 or 13 days, and the final length of buds in each explant is shown with a black dot, n = 41–80. (B) Relative growth index (RGI) for the data in panel A. The black dot indicates the mean. The RGI is calculated as the length of the longest branch divided by the summed length of both branches. Only active explants are used to calculate the RGI. Active explants are those with at least one active bud (a bud is considered active if it has a maximum growth rate > 2.5 mm/day), n = 12–80. (C) Percentage of explants with at least one active bud in each of the three experimental replicates. (D) Maximum growth rate and (E) Number of lag days for the longer buds in panel A. The number of lag days is calculated as the number of days before buds reach a growth rate of 2.5 mm/day. The data are plotted for active buds only, n = 12–80. In panels B–E, S1p1p7 and S1p1b1b2 are used as shorthands for SIP1pin1pin7, and SIP1pin1brc1brc2, respectively. Asterisks indicate statistically significant differences at p < 0.05 from a multi-level model with custom contrast on the comparisons of interest and Bonferroni corrections. Data and analysis underlying this figure can be found at doi 10.17863/CAM.120831. (TIF) [file pbio.3003395.s008.tif]
