# Supplementary material for: A BRC1-modulated switch in auxin efflux accounts for the competition between Arabidopsis axillary buds
Source: PLoS Biol. 2025 Sep 15;23(9):e3003395. doi: 10.1371/journal.pbio.3003395 (PMC12456834; doi:10.1371/journal.pbio.3003395)
Supplement: Attachment — Submitted filename: PBIOLOGY-D-25-01168R1_response_reviewers.docx [file pbio.3003395.s012.docx]

Dear Ines,

We are pleased to submit our revised manuscript for publication in PLOS Biology.

We are grateful for your comments, and those of the two reviewers, which have helped improve the manuscript. We have revised the manuscript to address all the points raised. The changes made are described below. We have also addressed the technical requests and respond to them at the end of the document.

In addition, we have added two brief clarifications to the appendix: one explaining that v0 and μ are independent parameters in the final model (page 3), and another clarifying that, for simplicity, the derivation omits scaling constants that would resolve any apparent dimensional inconsistencies (page 5). These additions improve clarity without affecting the model’s results or conclusions.

In making all the final double checks for publication, we found 2 very minor errors which we have now corrected. First, we noticed a minor error in the code to generate figures S6 and S7E. This modifies very slightly the statistics of *smxl678* big buds in fig S6. Second, we found a minor error in the code for figure 4B(ii) which very slightly alters the yellow zone area. In both cases the differences are extremely small and do not impact any of the conclusions drawn.

We have included a version of the manuscript and appendix with all the changes highlighted. Thank you for considering our resubmission.

With best wishes,

Ottoline Leyser & James Locke

**Reviewers' comments**

Rev. 1:
This manuscript studies how auxin transport and the transcription factor BRC1 interact to regulate axillary bud outgrowth. Both responses are controlled by strigolactone (SL) signaling. Using two-node explant bud competition experiments in several genetic backgrrounds -brc1brc2, smxl678, and the brc1brc2 smxl678- and in response to GR24 (synthetic SL) treatments the authors investigate in depth how buds compete and respond, and how SL, BRC1 and the auxin. Transporter PIN1 participate in this process. These experiments allow them to generate and validate, also in detail, a mathematical model aimed at understanding the molecular components influencing the lag phase and the rapid outgrowth phase of bud activation. The extensive data, together with previous work from the same group, shows that SL acts through both a BRC1/2-dependent and a BRC1/2-independent pathway during bud outgrowth. In particular, during the rapid outgrowth phase, SL appears to influence auxin transport in a BRC1-independent manner.

The authors also design an elegant experiment, in which a domain of PIN1 is replaced with the corresponding domain of PIN3 to generate a modified PIN1 less responsive to SL (SIP1). The authors show that SIP1 protein is indeed more resistant to SL. However, interpretation of these results becomes confusing due to a partial SL sensitivity of the protein, noticeable in the response of buds to SL, and to impaired auxin transport in the SIP1 lines. This complicates the interpretation of all the experiments performed with this line (eg. Figure 8, Figures S6, S9).

We thank the reviewer for their help in improving the clarity of our manuscript. Indeed, as we described, PIN1 may have remaining strigolactone sensitivity independent of the L2 region, and the SIP1 line has impaired auxin transport. These are distinct points. We have clarified and reorganised the relevant discussion section to make each point separately, and discuss how we have incorporated them into our interpretation of the results (836-877). To improve clarity, we have also made small edits in the relevant results section (L.489, 548, 585), including changing the title of the final section (L. 588).

One of the main conclusions of the paper, also featured in the title of the manuscript, is that BRC1 modulates auxin transport. However, this point is not directly demonstrated. It is derived from interpretations of the modeling outputs and the behavior of buds in the competition assays. In the discussion, the authors note this limitation and acknowledge that no auxin transporters are currently known to be transcriptional targets of BRC1. They suggest a possible post-translational mechanism, though this remains speculative. Besides, one known BRC1 target, GH3.5, reduces active IAA levels through conjugation, which could offer an alternative explanation (not considered) by which BRC1 impacts auxin availability rather than transport per se.

We thank the reviewer for this important point. Our model is formulated in terms of auxin efflux. The auxin efflux from a bud depends on the amount of auxin exported via transporters and diffusion, both of which are influenced by auxin levels (explained and now clarified in L. 235-237). Our model is consistent with BRC1 modifying basal auxin efflux (as stated in L.37, 721, 819), rather than specifically auxin transporters. We have modified the title of our manuscript to clarify this point. This could indeed occur by regulating available auxin concentration, consistent with the reviewer’s suggestion. We have clarified further the role of BRC1 and the formulation of auxin efflux when presenting the model (L. 222, 248, 303-306), and have expanded the relevant section of the discussion to incorporate the hypothesis suggested (L. 775-777, 809-817). Furthermore, to be more specific we now state that BRC1 may downregulate the basal rate of efflux rather than tuning it (L. 37, 759).

Auxin transporter activity and auxin levels are deeply intertwined. For example, feedback on both auxin synthesis and auxin conjugation typically maintain auxin at a homeostatic level. Therefore, if auxin is transported away from a site of synthesis, more will be made and/or less will be conjugated at that site. The match between model and experiment provides a strong justification for future work to test directly for changes to auxin export, through changes in auxin transporter activity and/or auxin metabolism.

It is also worth noting that while all genetic experiments are carried out using the brc1 brc2 double mutant, the qPCR analyses and the modeling (and the title) focus on BRC1. This raises the question of why is BRC2 excluded from these parts of the study.

The modest branching phenotype of *brc2* compared to *brc1*, including the observation that the *brc1brc2* double mutant has the same number of branches as *brc1* (Aguilar-Martinez et al., 2007), suggests that BRC2 has a more minor role in shoot branching regulation. We use the double mutant because there may be some redundancy between the two genes, but it is reasonable to assume that results from experiments using *brc1brc2* can be more heavily attributed to BRC1 function. Nonetheless, we agree that a minor role of BRC2 cannot be excluded. We have clarified this point in the text (L. 166-170).

Rev. 2:
The manuscript by Nahas et al is an elegant integration of computational modeling and experimental validation—both aimed at formalizing a minimal regulatory model for competition between buds. The authors have done a significant amount of work, and it is all thoughtfully designed, appropriately controlled and interpreted with reasonable cautions. The engineering of a largely SL-independent PIN1 was excellent, although more work could be done to enhance interpretability, as the authors describe. Available mutants are used to great effect throughout.

The utility of a model with minimal parameters cannot be overstated, especially for hypothesis generation, and the authors have done an admirable job of capturing decades of findings within a strikingly simple architecture. Essentially, the authors argue that both model and validating experiments point to two significant tuning knobs that together determine whether one or both buds will grow in a 2-node explant: (1) how much auxin efflux transporter is removed from the membrane, and (2) what is the basal rate of auxin efflux. They go on to show that strigolactones primarily control the former, while BRC1 is largely acting on the latter.

I have no major concerns, but a few questions:

1) If I am reading the model correctly, there is no parameter that is dependent on the amount of auxin in the system. If I understand that correctly, I wonder if that partially explains the unexpected behavior of the smxl678 mutant in the 2 node vs 1 node assay. The authors argue that sugar could be playing a role here, and I wonder if that effect could be by boosting the total level of auxin in the system. If there was more overall auxin, perhaps both buds could reach the 'tipping point' of canalization more quickly.

We thank the reviewer for this interesting suggestion. The concentration of auxin in the system appears in the model derivation, but in order to obtain a simple model, as described in response to referee 1, we combine auxin concentration and transporter concentration into the auxin efflux term E.

Higher global auxin concentration in the model (see mathematical appendix, page 3), would increase the values of parameters v (maximum rate of the Hill function) and v0 (basal rate of auxin efflux). Higher values of v0 and v increase the probability of bud activation (Fig S2). Therefore, our model agrees with the hypothesis of this reviewer, and we have expanded the text to include it (L. 922-927). This is also consistent with current hypotheses for the mode of sugar action in bud regulation, which includes that sugar promotes auxin synthesis.

2) It is a personal preference, but in the interest of readability, I would love to see fewer abbreviations used throughout the text. Maximum growth rate is somewhat cumbersome to spell out, but MGR is not really an improvement in my opinion. Similarly, in Figures like S1, it would be wonderful to have the meanings of each parameter being tested spelled out in the titles of each graph or at least in the figure legend to avoid having to flip back and forth.

Figure S1 has been amended to include parameter definitions, and panels have been reordered to match the order of the parameter definition in the text. To reduce the number of abbreviations, we have removed MGR and now spell out maximum growth rate throughout the text and in the figures (fig 6, 9, S4, S8). For the relative growth index (RGI), we respecify the meaning of the abbreviation at the beginning of the results, discussion, and in each figure legend. Overall, we hope these changes improve the readability of the manuscript.

3) It would be useful to have the authors speculate a bit on how the current model might succeed or struggle in a more complex system of higher numbers of buds, and other considerations that might need to be incorporated to apply the model to other plants (e.g., different shoot architectures).

We thank the reviewer for this suggestion. We agree that these are important points and have added a paragraph to the discussion to include them (L. 767 - 771).

**Journal Requirements:**

1) Your current Financial Disclosure states, “ ZN, AB, FT, JD, MS, OL were supported by the Gatsby Charitable Foundation No. GAT3272C awarded to OL. KA, DCB, TL, JL were supported by Gatsby Charitable Foundation No. GAT3395/PR5 awarded to JL, with the work in JL lab also supported by BBSRC grants award no. BB/V006088/1 and BB/X020126/1. ”. 

However, your funding information on the submission form indicates that you did not receive funding. Please indicate by return email the full and correct funding information for your study and confirm the order in which funding contributions should appear. Please be sure to indicate whether the funders played any role in the study design, data collection and analysis, decision to publish, or preparation of the manuscript.

We can confirm that the information in the financial disclosure is correct.

2) Please amend your detailed Financial Disclosure statement. This is published with the article, therefore should be completed in full sentences and contain the exact wording you wish to be published.
i). State what role the funders took in the study. If the funders had no role in your study, please state: “The funders had no role in study design, data collection and analysis, decision to publish, or preparation of the manuscript.”
ii). If any authors received a salary from any of your funders, please state which authors and which funders.

Done.

3) We ask that a manuscript source file is provided at Revision. Please upload your manuscript file as a .doc, .docx, .rtf or .tex.

Done.

4) Please provide separate figure files in .tif or .eps format.

For more information about figure files please see our guidelines: 

<https://eur03.safelinks.protection.outlook.com/?url=https%3A%2F%2Fjournals.plos.org%2Fplosbiology%2Fs%2Ffigures&data=05%7C02%7Czoe.nahas%40slcu.cam.ac.uk%7C4f6d494a901f4952799208ddacb65b0b%7C49a50445bdfa4b79ade3547b4f3986e9%7C1%7C0%7C638856621433452734%7CUnknown%7CTWFpbGZsb3d8eyJFbXB0eU1hcGkiOnRydWUsIlYiOiIwLjAuMDAwMCIsIlAiOiJXaW4zMiIsIkFOIjoiTWFpbCIsIldUIjoyfQ%3D%3D%7C0%7C%7C%7C&sdata=6VBpNtbjjclo2qmBbMKgdCjWmG3OkpnXM%2FF3OG%2BLjN0%3D&reserved=0>  

<https://eur03.safelinks.protection.outlook.com/?url=https%3A%2F%2Fjournals.plos.org%2Fplosbiology%2Fs%2Ffigures%23loc-file-requirements&data=05%7C02%7Czoe.nahas%40slcu.cam.ac.uk%7C4f6d494a901f4952799208ddacb65b0b%7C49a50445bdfa4b79ade3547b4f3986e9%7C1%7C0%7C638856621433480126%7CUnknown%7CTWFpbGZsb3d8eyJFbXB0eU1hcGkiOnRydWUsIlYiOiIwLjAuMDAwMCIsIlAiOiJXaW4zMiIsIkFOIjoiTWFpbCIsIldUIjoyfQ%3D%3D%7C0%7C%7C%7C&sdata=xTdEOwwlSp%2Bxb5YyjCyltYIqd2HsGvD0Rkl1R8T3zGY%3D&reserved=0>

We have submitted all figure files in .tif format.

5) Please upload a copy of Figure [1 - 9] which you refer to in your text on your manuscript. Or, if the figure is no longer to be included as part of the submission please remove all reference to it within the text.

We are unsure what is requested here as we could not find reference to Figure [1 – 9] in the manuscript. Please let us know if this is still a concern?

6) We notice that your supplementary [figures] are included in the manuscript file. Please remove them and upload them with the file type 'Supporting Information'. Please ensure that each Supporting Information file has a legend listed in the manuscript after the references list.

Done.

7) We have noticed that you have uploaded Supporting Information files, but you have not included a list of legends. Please add a full list of legends for your Supporting Information files after the references list. 

Done.

8) We have noticed that you have cited " Supplementary Figure S4 " in your manuscript. However there are no corresponding files uploaded to the submission. please upload them as separate files with the item type supporting information.

Thank you for spotting this. We have corrected the Supplementary Figure order.

9) Thank you for your statement " All data and code will be made available via the University of Cambridge data repository. " 

In your Data Availability Statement, please ensure you have provided all necessary access information in complete sentences. Access information should include the name of any data repositories used as well as digital object identifiers (DOIs), accession numbers or codes, or other persistent identifiers for all relevant data.

Complete data and code will be made accessible on the Cambridge university data repository upon acceptance of the manuscript. We will add the DOI at the proof stage.

10) Some material included in your submission may be copyrighted. According to PLOS’s copyright policy, authors who use figures or other material (e.g., graphics, clipart, maps) from another author or copyright holder must demonstrate or obtain permission to publish this material under the Creative Commons Attribution 4.0 International (CC BY 4.0) License used by PLOS journals. Please closely review the details of PLOS’s copyright requirements here: PLOS Licenses and Copyright. If you need to request permissions from a copyright holder, you may use PLOS's Copyright Content Permission form.

Please respond directly to this email and provide any known details concerning your material's license terms and permissions required for reuse, even if you have not yet obtained copyright permissions or are unsure of your material's copyright compatibility. Once you have responded and addressed all other outstanding technical requirements, you may resubmit your manuscript within Editorial Manager. 

Potential Copyright Issues:

1) "[Figure 1c, 2b,c]: Please confirm whether you drew the images / clip-art within the figure panels by hand. If you did not draw the images, please provide (a) a link to the source of the images or icons and their license / terms of use; or (b) written permission from the copyright holder to publish the images or icons under our CC BY 4.0 license. Alternatively, you may replace the images with open source alternatives. See these open source resources you may use to replace images / clip-art:
- <https://eur03.safelinks.protection.outlook.com/?url=https%3A%2F%2Fcommons.wikimedia.org%2F&data=05%7C02%7Czoe.nahas%40slcu.cam.ac.uk%7C4f6d494a901f4952799208ddacb65b0b%7C49a50445bdfa4b79ade3547b4f3986e9%7C1%7C0%7C638856621433495188%7CUnknown%7CTWFpbGZsb3d8eyJFbXB0eU1hcGkiOnRydWUsIlYiOiIwLjAuMDAwMCIsIlAiOiJXaW4zMiIsIkFOIjoiTWFpbCIsIldUIjoyfQ%3D%3D%7C0%7C%7C%7C&sdata=%2F2EtZxHtxnpa08rxFm28vNxM8ZFMjw7eZNYKRPSeRF4%3D&reserved=0>
- [https://eur03.safelinks.protection.outlook.com/?url=https%3A%2F%2Fopenclipart.org%2F&data=05%7C02%7Czoe.nahas%40slcu.cam.ac.uk%7C4f6d494a901f4952799208ddacb65b0b%7C49a50445bdfa4b79ade3547b4f3986e9%7C1%7C0%7C638856621433509380%7CUnknown%7CTWFpbGZsb3d8eyJFbXB0eU1hcGkiOnRydWUsIlYiOiIwLjAuMDAwMCIsIlAiOiJXaW4zMiIsIkFOIjoiTWFpbCIsIldUIjoyfQ%3D%3D%7C0%7C%7C%7C&sdata=J8Ek%2BXGz9JJXBbpXgrxuYJ9WYP7VohjwbWWRt7CxdUw%3D&reserved=0"](https://eur03.safelinks.protection.outlook.com/?url=https%3A%2F%2Fopenclipart.org%2F&data=05%7C02%7Czoe.nahas%40slcu.cam.ac.uk%7C4f6d494a901f4952799208ddacb65b0b%7C49a50445bdfa4b79ade3547b4f3986e9%7C1%7C0%7C638856621433509380%7CUnknown%7CTWFpbGZsb3d8eyJFbXB0eU1hcGkiOnRydWUsIlYiOiIwLjAuMDAwMCIsIlAiOiJXaW4zMiIsIkFOIjoiTWFpbCIsIldUIjoyfQ%3D%3D%7C0%7C%7C%7C&sdata=J8Ek%2BXGz9JJXBbpXgrxuYJ9WYP7VohjwbWWRt7CxdUw%3D&reserved=0%22)

We drew these figures by hand using Adobe Illustrator. We now indicate this in the figure legend.

11) "Please refer to the following article for PLOS Biology's best-practice recommendations for data presentation: <https://eur03.safelinks.protection.outlook.com/?url=https%3A%2F%2Fjournals.plos.org%2Fplosbiology%2Farticle%3Fid%3D10.1371%2Fjournal.pbio.1002128&data=05%7C02%7Czoe.nahas%40slcu.cam.ac.uk%7C4f6d494a901f4952799208ddacb65b0b%7C49a50445bdfa4b79ade3547b4f3986e9%7C1%7C0%7C638856621433522796%7CUnknown%7CTWFpbGZsb3d8eyJFbXB0eU1hcGkiOnRydWUsIlYiOiIwLjAuMDAwMCIsIlAiOiJXaW4zMiIsIkFOIjoiTWFpbCIsIldUIjoyfQ%3D%3D%7C0%7C%7C%7C&sdata=iFf%2Btu9SKf9wXNsZzIgn7iF8d8DY5hbACXfQbyX0xrs%3D&reserved=0>.

Guidelines for statistical reporting, if relevant, may be found here: [https://eur03.safelinks.protection.outlook.com/?url=https%3A%2F%2Fjournals.plos.org%2Fplosbiology%2Fs%2Fbest-practices-in-research-reporting%23loc-statistical-reporting&data=05%7C02%7Czoe.nahas%40slcu.cam.ac.uk%7C4f6d494a901f4952799208ddacb65b0b%7C49a50445bdfa4b79ade3547b4f3986e9%7C1%7C0%7C638856621433536919%7CUnknown%7CTWFpbGZsb3d8eyJFbXB0eU1hcGkiOnRydWUsIlYiOiIwLjAuMDAwMCIsIlAiOiJXaW4zMiIsIkFOIjoiTWFpbCIsIldUIjoyfQ%3D%3D%7C0%7C%7C%7C&sdata=h5to8uNd%2BMlboRPYn2ei3e%2Bwte7%2FAkZcYbVBTwiuCfs%3D&reserved=0."](https://eur03.safelinks.protection.outlook.com/?url=https%3A%2F%2Fjournals.plos.org%2Fplosbiology%2Fs%2Fbest-practices-in-research-reporting%23loc-statistical-reporting&data=05%7C02%7Czoe.nahas%40slcu.cam.ac.uk%7C4f6d494a901f4952799208ddacb65b0b%7C49a50445bdfa4b79ade3547b4f3986e9%7C1%7C0%7C638856621433536919%7CUnknown%7CTWFpbGZsb3d8eyJFbXB0eU1hcGkiOnRydWUsIlYiOiIwLjAuMDAwMCIsIlAiOiJXaW4zMiIsIkFOIjoiTWFpbCIsIldUIjoyfQ%3D%3D%7C0%7C%7C%7C&sdata=h5to8uNd%2BMlboRPYn2ei3e%2Bwte7%2FAkZcYbVBTwiuCfs%3D&reserved=0.%22)

We have followed the above guidelines, except we do not currently indicate the exact p-values throughout the manuscript. This is cumbersome in the case of multiple pairwise comparisons, which we use recurringly. If needed, we could submit this information as supplementary tables.
