# Supplementary material for: A BRC1-modulated switch in auxin efflux accounts for the competition between Arabidopsis axillary buds
Source: PLoS Biol. 2025 Sep 15;23(9):e3003395. doi: 10.1371/journal.pbio.3003395 (PMC12456834; doi:10.1371/journal.pbio.3003395)
Supplement: Attachment — Submitted filename: PBIOLOGY-D-25-01168R2_Data_policy_compliance.docx [file pbio.3003395.s013.docx]

Dear Ines,

Thank you for all your help with our manuscript, PBIOLOGY-D-25-01168R2, which we have amended to ensure it complies with the PLOS Biology data policy.

All the data and code underlying its analysis has been deposited in the Cambridge University data repository with doi [10.17863/CAM.120831](https://doi.org/10.17863/CAM.120831). We now refer to this in each figure legend.

We have reviewed the references, and noticed a few instances where publications were referenced in the text but were missing from the bibliography. This has now been fixed. We have also corrected and/or added information in the bibliography where necessary. Additionally, we discovered minor errors in two of the supplementary figures (S3 and S8), which we have corrected. These changes are extremely small and have no impact on the conclusions, as can be seen from the corrected versions. We have included a version of the manuscript and supplementary figures with all changes highlighted.

Thank you again for your help.

With best wishes,

Ottoline Leyser & James Locke
